# Thermodynamic States in Nonhomogeneous Systems: From Nanoscale to Macroscale

**DOI:** 10.1021/acsomega.4c11379

**Published:** 2025-04-09

**Authors:** Sankhadeep Bose, Andrea Floris, Mangaiyarkarasi Rajendiran, Bruno D’Aguanno

**Affiliations:** † School of Mechanical Engineering, Vellore Institute of Technology, Vellore 632014, Tamil Nadu, India; ‡ Department of Chemistry, School of Natural Sciences, University of Lincoln, Brayford Pool, LN6 7TS Lincoln, U.K.; § Centre for Nanotechnology Research, Vellore Institute of Technology, Vellore 632014, Tamil Nadu, India

## Abstract

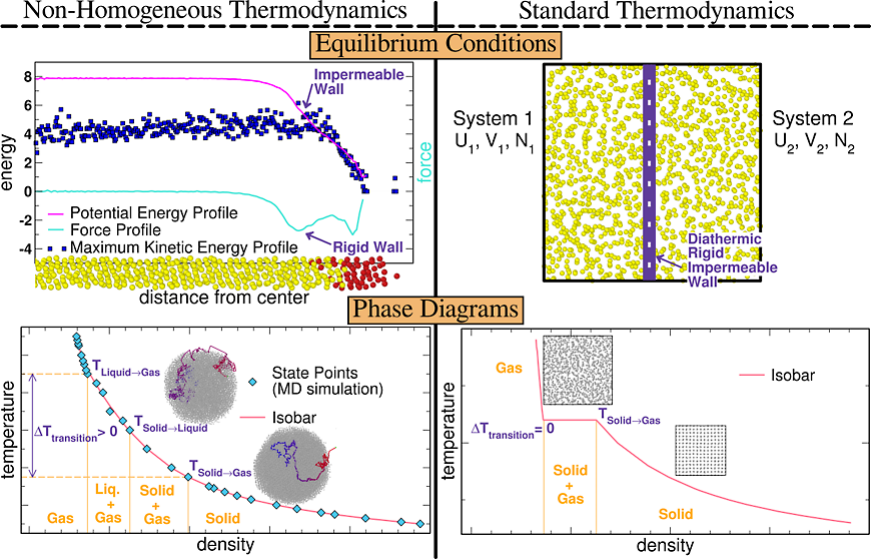

We analyze the mechanisms leading to thermodynamic stable states and isobaric phase transitions in finite nonhomogeneous nanosystems using classical molecular dynamics. We consider systems ranging from nano- to macroscopic scales and focus on spherical Lennard-Jones nanoparticles, in both one- and two-phase equilibria. In particular, we investigate how these systems’ macroscopic behaviors evolve as their size increases. Our findings unveil that nonhomogeneous stable states are governed by spatial variations in intensive variables, contrary to standard thermodynamics of homogeneous systems, where equilibrium is described by extensive variables. Crucially, we demonstrate that nonhomogeneous intensive variables persistently diverge from homogeneous systems’ predictions, even as the system size increases. Our calculations show that one-phase equilibrium is the direct consequence of the spatial variations of these intensive variables. In the two-phase equilibrium, such variations generate isobaric phase transitions across *finite* temperature intervals, through a continuous sequence that includes three-phase states. These temperature ranges *do not* vanish with increasing size, challenging the assumption that homogeneous systems are the asymptotic limit of finite nonhomogeneous systems. Our findings highlight the significance of boundary effects in understanding thermodynamic stability and equilibrium mechanisms, marking a departure from standard thermodynamic models that neglect these variations.

## 1 Introduction

Finite nonhomogeneous systems are macroscopic structures characterized by an interfacial region, when in contact with another material or when in vacuum. They encompass all real liquids and solids as well as real gases in proximity to a phase transition, where interfacial regions are well characterized. Although less pronounced, these regions are also present in finite-size semiclassical ideal gases,^1^ while they are absent in finite ideal gases enclosed in an inert container, as they are inherently homogeneous.^1^

In this work, we will contrast finite nonhomogeneous systems (FNHs) with homogeneous systems, which do not have boundaries and are described by standard thermodynamics. Via classical molecular dynamics (MD), we will analyze the stability conditions in one- and two-phase FNHs, highlighting the discrepancies between their thermodynamics and those of homogeneous systems. We will demonstrate that, in many respects, these nonhomogeneous systems do not converge to their homogeneous counterparts even in the limit of very large system size. To show this, we will consider spherical Lennard-Jones nanoparticles characterized by an inner homogeneous region with uniform physical properties, and by an outer, nonuniform surface region (interface). These are simple prototypes of real solids or liquids, which in general can have more complex structures/boundaries.

By standard thermodynamics (ST), we refer specifically to the axiomatic formalism developed by Callen^3^ and related works.^4−8^ This theory considers composite systems characterized by internal constraints and, among other considerations,^2^ it assumes the homogeneity of each subsystem.^3−6,8^ As such, ST is a model of real systems, which we refer to as the ST model. Although very general, the model has its own range of applicability, and its results are dependent on the assumptions of the model itself. Several postulates characterize the model, with the following three being central to our discussion:

1. Equilibrium states are particular states that are completely characterized, macroscopically, by the internal energy *E* and by a small set of extensive parameters ***X*** which are specific to the system.
2. There is a function of state, the entropy *S*(*E*, ***X***), such that the values of ***X*** assumed in an isolated composite system in the absence of internal constraints are those that maximize *S* over the set of constrained equilibrium states.
3. The entropy of a composite system is additive over the constituent subsystems. *S* is a continuous and differentiable function and is a monotonically increasing function of the internal energy *E*.

In homogeneous systems, the additivity of *S* implies its extensivity; i.e., the homogeneity assumption implies the extensivity of *S*.^3^ By virtue of postulate 3, in these systems, the extensivity of *S* also implies the extensivity of *E*.^3,4,7^ Conversely, if a system is characterized by macroscopic variables (*S*, *E*, ***X***) which are not extensive, then the system is nonhomogeneous. In FNHs with interfacial regions, *S* and *E* (and other thermodynamic potentials) will not be in general extensive, and deviations from corresponding homogeneous values are expected. Some deviations are well known, e.g., in the heat capacity *C*(9) or whenever (as in the spheres considered here) volumes of bulk and interfacial regions scale differently with the number of particles (as *N* and *N*^2/3^, respectively).^10^ These deviations are relevant for small systems but become very small and eventually vanish in the thermodynamic limit, where the homogeneous values of many macroscopic variables are recovered.^11,12^ Hence, large FNHs will have the same values of macroscopic variables, as the corresponding homogeneous systems.

This work, however, will first show that some differences, concerning *intensive* variables, are not eliminated for very large *N* or in the thermodynamic limit. Specifically, after defining suitable radial profiles of intensive variables, we will show that in FNHs, these variables become *spatially dependent*, unlike in homogeneous systems.^4^ This spatial dependence persists even for *N* → ∞, making it an intrinsic feature of FNHs. Second, we will investigate the mechanisms that stabilize FNH systems, showing how the equilibrium requires the presence of intensive variables *gradients*, acting as “internal physical constraints”. These will be contrasted with the mathematical constraints necessary in standard thermodynamics to describe the equilibrium in homogeneous systems. A third aspect of the ST model which will be contrasted by our results is that, in *T*–*V* diagrams, isobars are characterized by a constant *T* during phase transitions (and in *p*–*V* phase diagrams, isotherms have a constant *p*). In FNHs, we will describe instead a sequence of processes, where the whole transitions occur during finite Δ*T* intervals which, crucially, *do not* tend to zero for *N* → ∞. Other transition temperatures, such as the sublimation temperature, also do not reach the corresponding homogeneous values in this limit. This demonstrates that “the homogeneous limit” is not a suitable approximation for real solids and liquids.

Analyzing the local thermodynamic properties of nonhomogeneous systems from nano to macro size is also a primary focus of nanothermodynamics. However, this work aims to provide a detailed analysis of the microscopic dynamical behavior of all the atoms in the NPs, and the analysis is based on MD “experimental” results obtained from a series of NPs of increasing size. Each NP consists of a collection of coupled thermodynamic subsystems (the radial shells introduced below). Therefore, we believe that our computational analysis is relevant for further development of theoretical approaches in nanothermodynamics, particularly those presented in refs (1,13–18).

The paper is organized as follows: we first introduce the MD parameters and setups for studying the Lennard-Jones (LJ) spheres. Section 2.1 describes the one-phase stable systems via the set of above-mentioned radial profiles. A finite chain of atoms is used as a preliminary tool to clarify the profile’s features for the more complex 3D nanoparticles. A detailed characterization of the stability in terms of the profiles will be followed by a discussion on how to reconcile our MD results with the standard thermodynamics model. Section 2.2 examines the equilibrium between two phases in contact with each other. We will investigate isobaric phase transitions in the spheres as a function of temperature, highlighting the deviations from homogeneous systems. Finally, in Section 3, we draw our conclusions, and in Section 4, we illustrate the methods used.

## 2 Results and Discussion

We will consider spheres where the interacting atoms experience the Lennard-Jones interaction potential *e*_LJ_(*r*) = 4ε[(σ/*r*)^12^ – (σ/*r*)^6^]. This potential has been widely used as a benchmark reference for all van der Waals materials^19,20^ and in molecular dynamics, statistical mechanics, and computational science studies. Moreover, materials well described by the LJ potentials also align with the assumptions of the ST model (see footnote^2^). We choose a spherical symmetry as it produces a large surface reconstruction (after the sphere is carved from an fcc crystal), making spherical systems excellent candidates for studying the effects of nonhomogeneity. Two simulation setups have been designed to investigate one- and two-phase equilibria (see Table 1).

**Table 1:** MD Simulation Setup^a^

MD setup	ensemble	box content	boundary conditions	reservoir	time step size
one-phase	*NVT*_eq_ → *NVT*_prod_	sphere at the center	fixed	Nose–Hoover chain (length 3)	0.005
two-phase	*NPT*_eq_ → *NVT*_eq_ → *NVT*_prod_	sphere at the center + inert gas	periodic	Nose–Hoover chain (length 3)	0.005, 0.05

a (...)_eq_ refers to equilibration step; (...)_prod_ refers to production step.

### 2.1 One-Phase Thermodynamic States

In this section, we will analyze the spheres in their one-phase stable state.^5^ Our atomic model parameters are reported in Table 2, while Table 3 illustrates the system parameters, where the nanoparticles are identified by their diameter (e.g., D30 refers to *d* = 30 diameter).^6^ The results of this section refer specifically to a D30 sphere at pressure *p* = 0.0, at two temperatures, *T* = 0.1 and *T* = 0.35.

**Table 2:** MD Model Parameters

MD model parameter	*m*	ε	σ	*r*_cut_ (shifted)^a^
sphere atoms	1	1	1	4
inert gas atoms	0.1	0.05	1	4
cross interactions^b^		0.2236	1	4

a A shifted potential *r*_cut_ = 4.0 is sufficient for the thermodynamic properties to converge due to the rapid *r*^–6^ decay, with an estimated discrepancy from the corresponding state law of less than 5%.^24−26^ See Section S4 for details.

b Parameters obtained from the Lorentz–Berthelot rules.

**Table 3:** MD System Parameters

MD system parameter	D30	D60	D90
number of sphere atoms	14713	115722	391285
number of inert gas atoms	40000	430000	1455000
*V*_Box_ (*T* = 0.3, *p* = 0.1)	166738	1556098	6031292
ρ_tot_ (*T* = 0.3, *p* = 0.1)	0.112	0.102	0.089

#### 2.1.1 Radial Profiles

**Figure 1:**
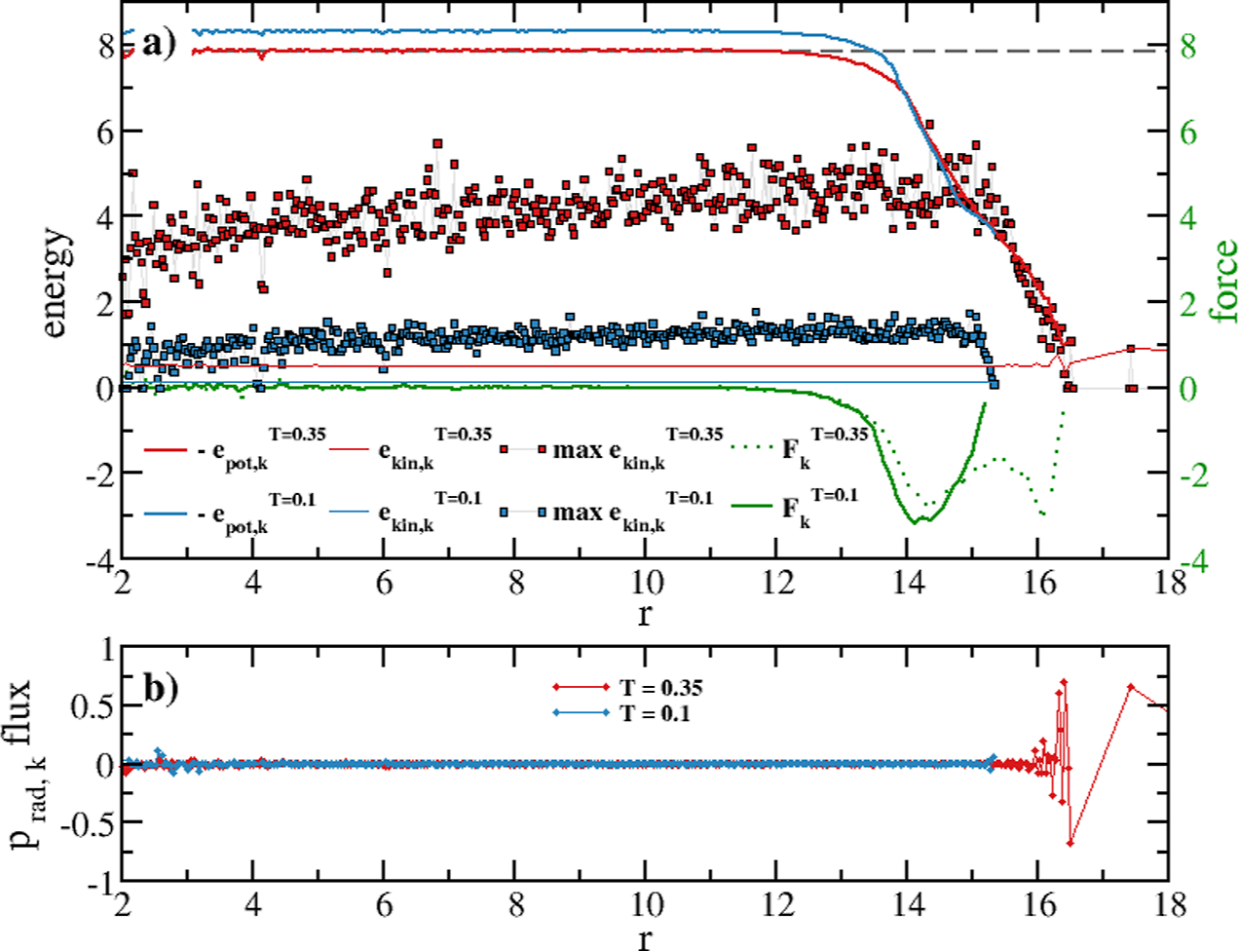
(a) Radial profiles of a D30 nanoparticle at T = 0.1 and T = 0.35, as a function of the distance from the sphere’s center. Potential energy: −*e*_pot,k_ (light-blue and red thick plots); the gray dashed line is the homogeneous system value at T = 0.35. Kinetic energy: max(*e*_kin*,*k_) (light-blue and red dots) and *e*_kin*,*k_ (light-blue and red thin lines). Force: *F*_k_ = −*de*_pot,k_/*dr*_k_ (full and dotted green plots). (b) Radial momentum flux *p*_rad,k_ profiles.

In Figure 1, starting from the sphere center, we plot a set of intensive variables radial profiles computed from MD trajectories. These plots are obtained by dividing the sphere into concentric shells labeled with the index k, with a corresponding radius *r*_k_ and a shell thickness δ*r* = *r*_k+1_ – *r*_k_.

As main quantities controlling the atom dynamics, we define the potential energy (*e*_pot,k_), kinetic energy (*e*_kin*,*k_), force (*F*_k_), radial momentum flux (*p*_rad,k_), and density (ρ_k_) profiles. These intensive quantities are computed at each *r*_k_ by using the equations in Table 4 (see also Section S1 for details).

**Table 4:** Intensive Variable Profiles Expressions (See Section S1 for Details)^a^

Profiles	MD relations
number density profile	ρ_k_ = ⟨*N*_k_⟩/Δ*V*_k_
diffusing atom fraction	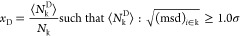
potential energy profile	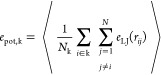
kinetic energy profile	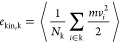
maximum kinetic energy profile	over all time steps
force profile	*F*_k_ = −d*e*_pot,k_/d*r*_k_
pair distance distribution	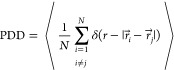

a *N*_k_ = ∑_*i*∈k_δ(*r* – *r*_*i*_) where k identifies the shell [*r*_k_, *r*_k_ + δ*r*] (*r*_k_ = 0 is the center of the sphere), , and ⟨...⟩ is the MD average.

The potential energy (PE) profile *e*_pot,k_ is an average, within the shell *r*_k_, of the LJ pair potentials between the particles in the shell and all particles in the system within a certain cutoff distance. Its negative values are plotted in Figure 1a and represent the energy barrier that atoms in the shell *r*_k_ must overcome, on average, to leave their potential wells. In the figure are also plotted the average kinetic energy (KE) profile *e*_kin*,*k_, and the maximum instantaneous KE values  among all atoms *i* in the shell *r*_k_ reached within the entire simulation time. Thus, if max(*e*_kin,k_) exceeds the (−*e*_pot,k_) PE, the atom has a probability to escape from its potential well; otherwise, it will remain trapped in it. As we will see, these radial profiles provide a 1D representation of the complex 3D energy landscape where the atoms vibrate or diffuse.

Some of these profiles have been widely used in the literature but have never been considered together, particularly in relation to atomic dynamics, to analyze the mechanisms leading to stability and equilibrium in both one- and two-phase states of nonhomogeneous real systems. Among intensive variable profiles, density is the most frequently analyzed. Density profiles have been studied for argon clusters^27^ (via Ar-parameterized LJ potential), glasses^28,29^ (via the Kob-Andersen LJ potential), silver–gold nanoparticles^30^ (from TEM experiments), and Si and C nanoparticles in molten salts.^9^ These studies consistently show that density profiles correspond to stable and well-equilibrated states. Pressure and density profiles have been analyzed for planar, cylindrical, and spherical interfaces,^31−33^ suggesting that the pressure profile and surface tension in well-equilibrated states are only weakly dependent on the surface curvature. Energy profiles, though less studied, have been investigated for stable LJ clusters,^27^ nanoglasses,^28^ and Au slabs.^34^ These studies showed a clear distinction between core and shell regions, with stability implying local equilibrium (and vice versa).

#### 2.1.2 Finite Chain of Atoms

**Figure 2:**
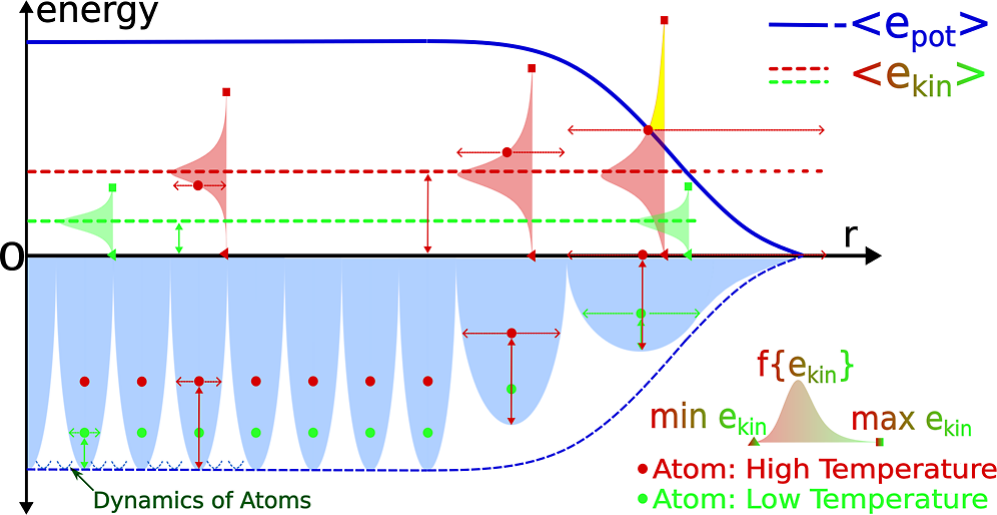
Sketch of the potential and kinetic energy profiles and distributions of a finite chain of atoms (see main text for details).

To clarify the meaning of the profiles in Figure 1, we first discuss the simple case of a truly 1D finite chain of atoms. The sketch illustrated in Figure 2, not derived from a MD simulation, contains all the elements needed to interpret correctly the nanoparticles profiles.

Along the chain, the PE changes continuously (light blue parabolas), becoming shallower and asymmetric when approaching the chain’s right end. In the upper part, the blue plot shows the negative values of the local PE minima. This plot is the analogue of the −*e*_pot,k_ plots in Figure 1a. Figure 2, upper part, also shows the atomic KE distributions, characterized by min (*e*_kin_) (triangles at zero energy), max(*e*_kin_) (square dots), and the average KE value (red and green dashed lines), at two temperatures. The distributions have different spreads, always maintaining a constant average. At low *T* (green atoms/plots), the average KE is low, with narrow distributions, essentially symmetric on approaching the chain end. Independent of the atomic position, no atom has an instantaneous max(*e*_kin_) to overcome the PE well where it moves. Thus, no atoms can leave the chain, which is then “geologically” stable (the chain’s volatility is zero).^35^ At high *T* (red atoms/plots), the average KE is higher, the distributions are more spread out, and their asymmetry increases near the chain end. In this case, the atomic position in the chain matters, and at the chain end, the atom has a finite probability of overcoming its PE well (yellow area). Hence, the atom might leave the chain (volatility is greater than zero) and, depending on the external pressure conditions, the system can either begin a solid-to-gas transition (*p* > 0) or disappear completely (p = 0).

The comparison between the PE profile and the instantaneous max(*e*_kin_) is the criterion to determine whether the system is (i) in a one-phase equilibrium (low *T*, any pressure); (ii) in a two-phase equilibrium (high *T*, *p* > 0); or (iii) unstable and undergoing a transition to another equilibrium phase (high *T*, p = 0). For chain lengths larger than the interaction range, it is reasonable to assume that at a given pressure *p*, the temperature *T* at which atoms start to leave the chain is independent of the number of atoms. This phenomenology should persist even for an extremely long finite chain (a FNH model), where the fraction of end-chain atoms approaches zero. Consequently, an infinite periodic chain (an infinitely large homogeneous model, ILH) will miss the described phenomenology, which is intrinsic to any real system with boundaries.

This preliminary example illustrates how the FNH model where *N* → ∞ and ILH model might have the same extensive variables, but their descriptions in terms of intensive variables differ, revealing evident deviations.

#### 2.1.3 Stability Mechanisms in Spherical Nanoparticles (NPs)

Returning to Figure 1a, and considering the blue plot in Figure 2, it is clear that −*e*_pot,k_ represents the convolution line of the local potential energy minima where the particles in each shell *r*_k_ sit. The horizontal portion of −*e*_pot,k_ indicates a homogeneous region inside the sphere, having the same PE as the corresponding homogeneous system. The PE then decays to the value of the farthest atom from the NP’s center and approaches zero at a distance outside the sphere. The *e*_pot,k_ spatial dependence implies a PE gradient, namely, the force *F*_k_ = −d*e*_pot,k_/d*r*_k_, which atoms experience due to the nonhomogeneous nature of the system (note that *F*_k_ = 0 in homogeneous systems) and the presence of interacting potentials (*F*_k_ = 0 in noninteracting systems). This force, intrinsic to every real FNH system, is the force required to evaluate the surface energy, the surface tension,^36−38^ and the pressure profile.^31−33^ It is always directed inward (*F*_k_ has negative values, Figure 1a, green plots) and has a temperature-dependent structure.

At T = 0.1 (solid green line), *F*_k_ exhibits a negative peak developing where the PE starts to deviate from a constant value, and it is zero in the inner sphere. At T = 0.35, *F*_k_ displays a double-peak structure (dotted green line), with the outer peak located in the NP’s most external region (for *r* ≈ 16).

In Figure 1a, the instantaneous KE max(*e*_kin,k_) values are also shown. The distribution *f*{*e*_kin*,*k_} broadens farther from the sphere’s center (especially at higher *T*), as indicated by a positive slope of max(*e*_kin,k_).^7^

At T = 0.1, the PE (blue solid line) is always larger than max(*e*_kin,k_) (light blue dots). This also holds near the surface region where the energy required is less. Thus, at this temperature, not a single atom has enough KE to leave the local PE minima. The D30 sphere has zero volatility and will remain stable, regardless of whether the sample is contained in a closed box or not (this is valid for classical systems where tunneling is not possible). T = 0.1 is then below the starting atom ejection temperature *T*_SG_.

By increasing the temperature to T = 0.35, the decrease of −*e*_pot,k_ and the increase of max(*e*_kin*,*k_) near the surface create the conditions for atoms to leave the surface. Specifically, to leave the nanoparticle, an atom must have (i) a max(*e*_kin*,*k_) > |*e*_pot,k_|; (ii) a radial outward momentum capable of overcoming the attractive force *F*_k_; (iii) no steric hindrance from other atoms (clearly accounted for in the LJ potential). At this temperature, atoms can leave the surface, and the NP acquires a finite volatility. If the sphere is not contained in a closed box, the number of atoms will decrease, the sample will not reach a stable state, and eventually it will sublimate completely. Our simulations indicate that the sublimation process initially follows a linear (slower) ejection rate, which then becomes nonlinear (faster) over time. This behavior has been consistently observed in experimental studies of Au^39,40^ and Ag^41−44^ nanoparticles. However, simulations for these materials have not been able to reproduce this behavior.^45−48^ If the NP is enclosed in a box, it will reach a point where there is an equilibrium between the two phases in contact, with the pressure in the simulation box corresponding to the gas pressure or gas tension.

**Figure 3:**
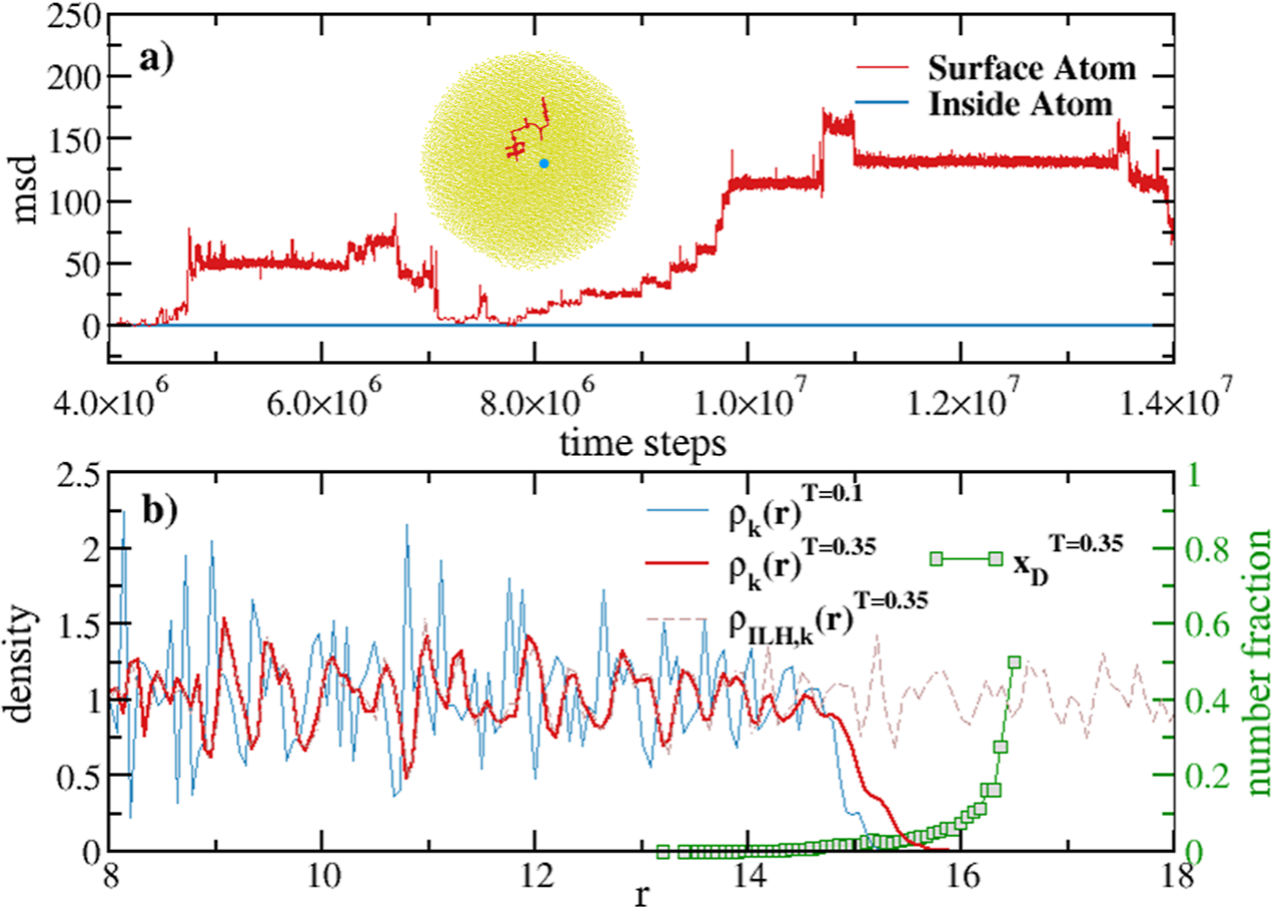
(a) Trajectories and mean squared displacements of a surface atom showing vibrational and diffusional motion (red line) and of an inner atom showing only vibrational motion (blue line/dot) in a D30 nanoparticle (NP). (b) Density profiles for a D30 NP and for the homogeneous system at different temperatures. The green squared plot *x*_D_ is a measure of the diffusional character of the atomic motion (see main text).

At both *T*’s, the nonhomogeneity in *e*_pot,k_ and *F*_k_ can also be considered as the generating factors for the nonhomogeneity in the density profiles, shown in Figure 3b. The strong oscillations in the inner part reflect the original fcc crystal structure (they are also present in the homogeneous system). As the surface is approached, this structure is first distorted, and then it disappears completely.

Additionally, by increasing the temperature, the KE distribution *f*{*e*_kin*,*k_}, despite generally broadening, becomes narrower within the interface region. We attribute this to a change in the nature of atomic motion, which in the solid inner part is purely vibrational (faster), while in the interface also acquires a diffusional character (slower), typical of a disordered or defective solid (as in this case) or a liquid. This observation is corroborated by computing the atomic mean square displacement msd. Figure 3b shows the number of atoms having .^8^ By choosing this value, only atoms with average vibration amplitudes larger than those in the inner region are singled out. Atoms with large msds are close to the sphere’s surface and are observed for T = 0.35 but not for T = 0.1. Their motion resembles that of disordered/defective solids or liquids, where atoms vibrate when confined in a deep PE cage,^53^ but occasionally, in regions where the cage is not that deep, they are able to jump into another cage, acquiring a diffusional motion.^53^ Here these regions are visible in Figure 3b, where the atom diffusion distribution  (green squares) represents the fraction of atoms in each shell k having a . Hence, our MD analysis is able to reproduce the atomic motion in terms of cages, as described in the seminal work of Frenkel^53^ and related works in the field of liquids.^54−56^

In Figure 1a, the red/light blue thin lines are KE averages ⟨*e*_kin,k_⟩. As sketched in the finite chain, the KE average is constant throughout the system. Consequently, the *T* profile must remain constant, meaning that the equilibrium temperature is the same everywhere, at both *T*’s (thermal equilibrium).

Finally, Figure 1b shows the radial momentum flux profile. At T = 0.1, this is zero everywhere, while at T = 0.35, it is not zero at the edge, due to the finite volatility.

In summary, at T = 0.1, despite the sphere being characterized by PE nonhomogeneity in *e*_pot,k_ and peaks in *F*_k_, chemical, thermal, and mechanical^9^ equilibrium are achieved everywhere.

The stability and local equilibrium conditions, as relevant indicators for nonhomogeneous systems, are thoroughly discussed in refs (35, 57, and 58). Stability is associated with the invariance of velocity and pair-distance probability distribution functions, while local equilibrium is indicated by the absence of net fluxes of momentum, energy, and mass. All our results satisfy the invariance of the profiles in time (and their underlying probability distribution functions) and show no net fluxes. Additionally, we find that fluctuations are larger in small systems, while relaxation times are shorter, as discussed in refs (35, 57, and 58).

#### 2.1.4 Molecular Dynamics and Standard Thermodynamics Model

These results outline the complexity of one-phase stability mechanisms in FNHs, in terms of MD radial profiles. Possibly, these conclusions could also be formulated in the language of the ST model for homogeneous systems, with the caveat of introducing formal (artificial) internal constraints, as opposed to the physical constraints arising from nonhomogeneity, which naturally account for the absence of mass, energy, and momentum fluxes at equilibrium.

In the ST model language, mechanical equilibrium (zero radial momentum flux) could be described by imagining the presence of a “rigid wall” capable of withstanding the surface tension (*F*_k_ per unit length). The wall would be located at the interface region where *F*_k_ ≠ 0 and its rigidity (basically the value of *F*_k_) continuously grow until ∇*F*_k_ ≈ 0, where |*F*_k_| is maximum. Thermal equilibrium (no net flux of energy) could be accounted for by the absence of adiabatic walls, indicating that the identified nonhomogeneity does not form such a wall or that its character is diathermal. Finally, chemical equilibrium (no flux of mass) could be described by imagining the presence of an impermeable wall preventing the atomic motion between regions with different *e*_pot,k_. The wall would be located in the region where −*e*_pot,k_ ≲ max(*e*_kin,k_), where the impermeability continuously grows until it becomes total at −e_pot,k_ ≳ max(*e*_kin,k_).

While these “walls” reconcile the two languages, it is worth noting that our MD simulations allow us to justify stability conditions without invoking any artificial constraints.

In summary, the thermodynamic stability conditions of FNHs are not governed by extensive variables but by the instantaneous and averaged values of intensive variables and their spatial dependence. Such probabilistic aspects of thermodynamic laws^35,59^ are completely absent from standard thermodynamics.

### 2.2 Two-Phase Thermodynamic States

**Figure 4:**
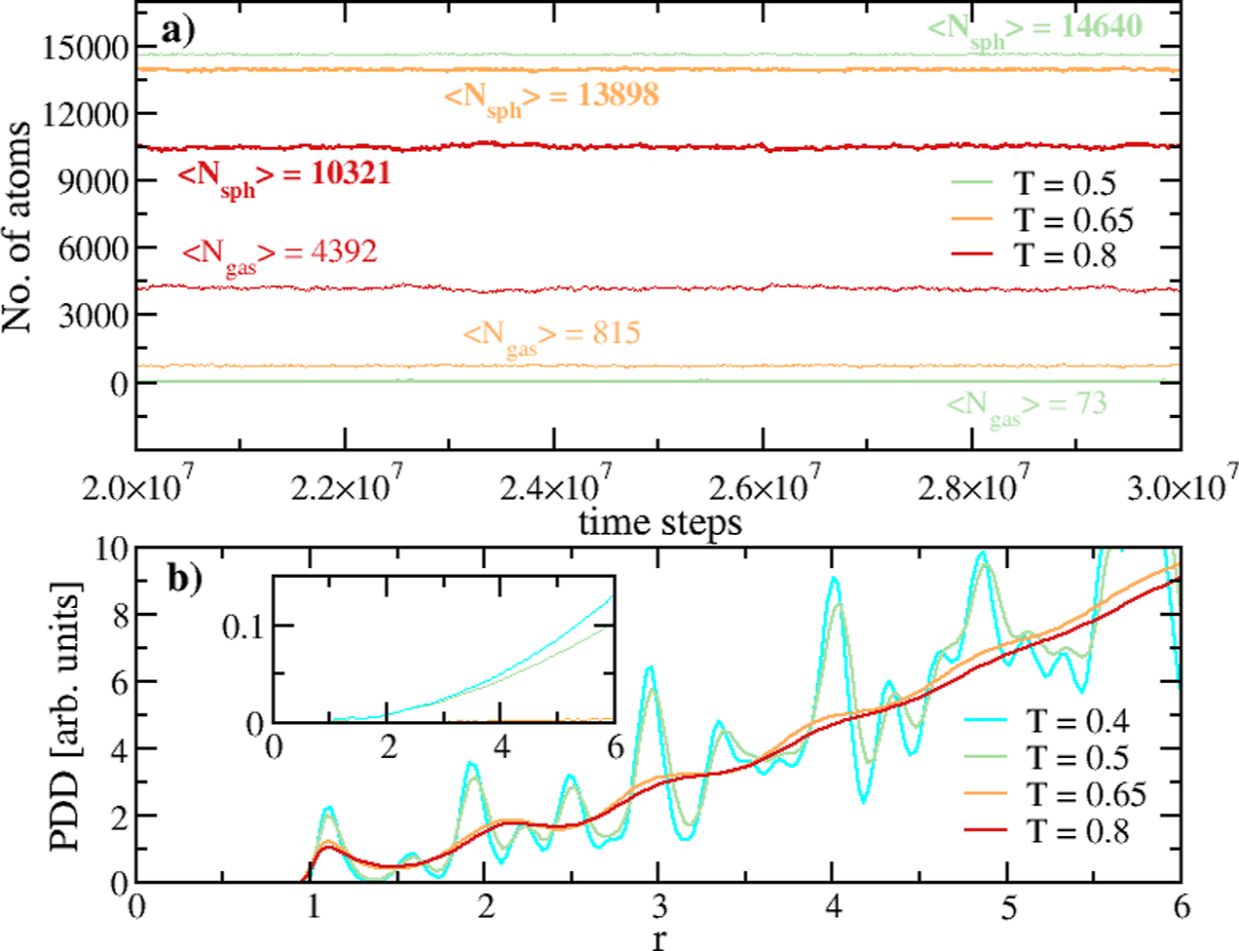
D30 nanoparticle data at p = 0.1 and different temperatures. (a) Number of atoms ⟨*N*_sph_⟩ in the sphere (thick lines) and in the gas phase ⟨*N*_gas_⟩ (thin lines) at equilibrium (T = 0.4 and T = 0.5 data superpose, only the latter are shown). (b) Pair distance distribution (PDD) function of the atoms in the sphere.

We will now discuss the two-phase equilibrium by analyzing a phase diagram during an isobaric phase transition. While in order to ascertain equilibrium states in one-phase, the best indicator is the system’s total energy (or other thermodynamic potentials requiring minimization), for two-phase equilibria, a more insightful indicator is the number of atoms in each coexisting phase, which, being the result of a dynamical process, will fluctuate over time around a constant value. This is shown in the horizontal plots of Figure 4a, demonstrating that the two-phase equilibrium has been carefully checked at each temperature at p = 0.1 (see also Section 4).

#### 2.2.1 Isobaric Transition of a D30 Nanoparticle

**Figure 5:**
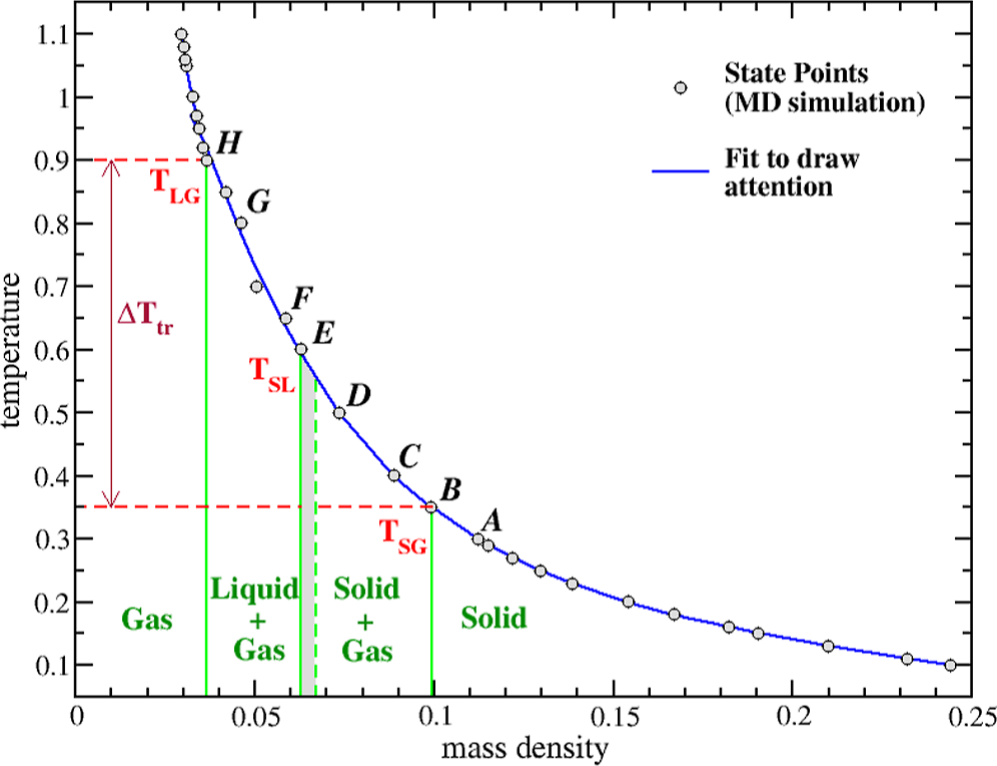
Phase diagram of a D30 nanoparticle inside an inert gas. The dots are MD calculations along an isobar at pressure p = 0.1. In the *x* axis, we report the system total mass density mN/V, which is changing with the volume V.

We will consider an isobaric process occurring to a FNH D30 sphere as a function of *T* at p = 0.1. The isobar is shown in Figure 5, which represents essentially a *T*–1/*V* phase diagram, where 1/*V* is plotted as average mass density after multiplication by the total constant mass mN present in the simulation box. As reported in Table 1, we perform a simulation in the *NPT* ensemble. Starting from a solid sphere (Figure 5, high mass density), we will follow its solid–gas transition by increasing the temperature, until the system is completely transformed into a gas (low mass density).

Using MD to analyze the two-phase equilibria at finite pressure technically requires the insertion, in the simulation box, of an “inert gas” able to sustain the chosen pressure (as in real experiments) and to avoid the interaction of the FNH system with its replica (if periodic boundary conditions are used, see Section 4). The presence of the inert gas is found to affect the quantitative results only marginally, while qualitative features are all preserved (see Section S2). The system can be thought of as staying in a closed container, where the two-phase equilibrium pressure corresponds to the gas pressure.

**Figure 6:**
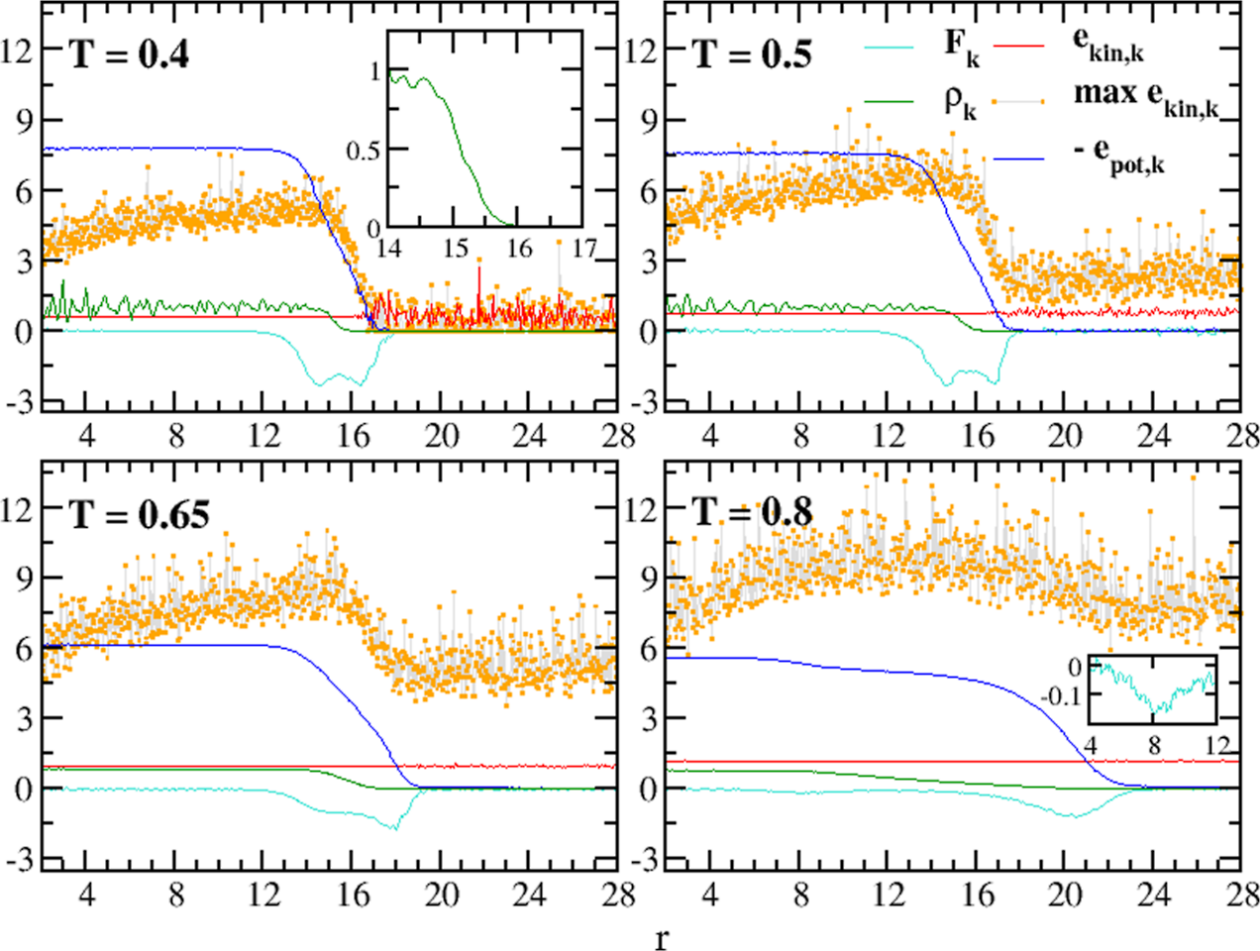
Radial profiles of a D30 nanoparticle (NP) at different *T*’s as a function of the distance from the NP center. Density ρ_k_ (green). Potential energy (−*e*_pot,k_) (light blue). Kinetic energy: max(*e*_kin*,*k_) (orange dots) and average ⟨*e*_kin,k_⟩ (red). Force *F*_k_ (cyan).

Figure 6 reports the spatially dependent intensive variables which control the system dynamical properties, as introduced in Section 2.1. First, one can see that the average KE (Figure 6, red lines) and the temperature profile are constant through the entire system. Temperature fluctuations are small, and they do not affect the atom’s dynamics at the interface.

Figure 5 shows that the stable state is a solid one-phase for T < 0.35 (point *A*), while in the interval T = 0.35–0.85, there is an equilibrium between two phases in contact (points *B*–*H*).

At T = 0.4, the strong oscillations in the density profiles (Figure 6, green plots) reveal a sphere solid core, as it is confirmed by the solid-like pair distance distribution (PDD)^10^ in Figure 4b (cyan plot). The core ends approximately at *r* ≈ 14. Here the atoms only vibrate; they cannot leave their potential wells, and there is no atom exchange with the outer region.

At T = 0.5 (Figure 5, point *D*), we find a similar behavior to that for T = 0.4. The higher *T* slightly increases the number of atoms in the gas phase; the inner region is smaller but remains solid (Figures 6 and 4b); the value of −(*e*_pot,k_) becomes slightly smaller, and its gradient region enlarges. At even higher *T*, we also observe in Figure 6 an evolution of the *F*_k_ minima, which shift to the right and become shallower, smoothing out due to a different exchange dynamics of the atoms at the interface.

**Figure 7:**
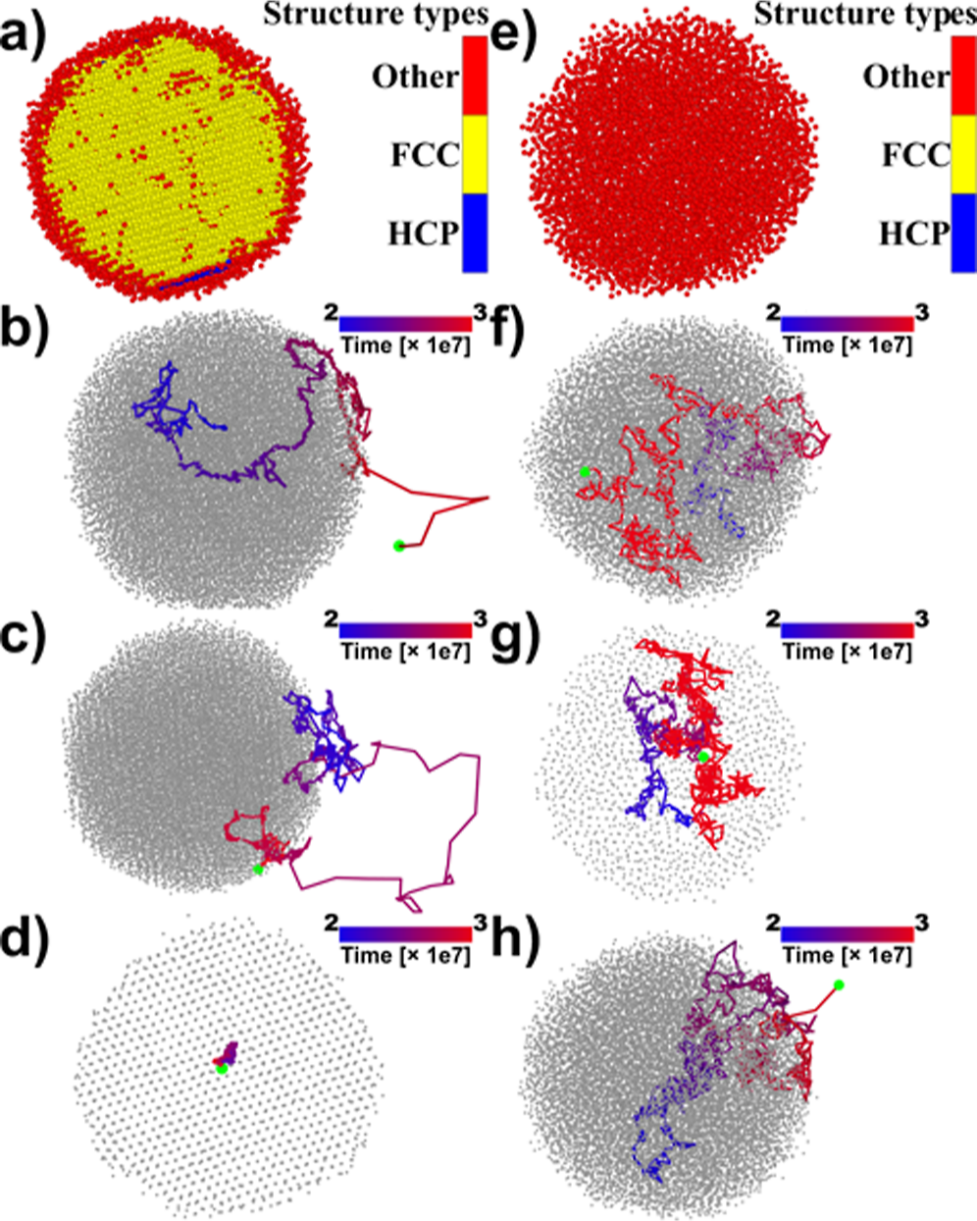
Snapshots of spheres at two temperatures. T = 0.5: (a–d); T = 0.65: (e–h).

The atomic behavior is also nicely illustrated by the MD trajectories over time. The snapshots in Figure 7a–d refer to T = 0.5. Panel (a) confirms that the interface atoms form a noncrystalline structure, while the inner atoms are in a fcc solid state. Interface atoms have a liquid-like diffusional behavior and are ejected after a long path in space and time (panel b). After a time proportional to the box size, the ejected atoms return to the interface region (panel c). Consequently, at T = 0.4–0.5, the interface liquid-like region is in equilibrium with both the inner solid core (panel d) and the outer gas, indicating effectively a three-phase region. The local structural environments in Figure 7a,e have been identified using the polyhedral template matching method.^61^

At T = 0.6 (Figure 5, point *E*), a qualitatively different behavior starts to emerge as the sphere becomes entirely liquid (points *E*–*G*). The density profile at a slightly higher T = 0.65 in Figure 6 does not show any oscillations in the core region (green line), similar to the PDD (Figure 4b, orange line), both typical of a liquid state. Hence, the system enters a two-phase, liquid–gas equilibrium. Panels (e–h) in Figure 7 refer to T = 0.65 and show that all atoms are noncrystalline (panel e) and that all sphere atoms exhibit a liquid diffusional behavior (panels (f) and (g) illustrate the trajectories of an atom from the interface and from the core, respectively). Moreover, atoms at the interface are ejected as at lower *T*’s (h). In view of this analysis, in going from T = 0.5 to T = 0.65, the system must have crossed a three-phase region or line, showcasing the absence of a triple point in FNH systems.

At T = 0.8 (Figure 5, point *G*), the liquid region becomes very small (where *e*_pot,k_ = const in Figure 6) and its density is very low. Although the KE have max(*e*_kin,k_) systematically larger than −*e*_pot,k_, the liquid core does not disappear. This is because a nonzero inward force *F*_k_ balancing the evaporation tendency is still present, with two minima in the profile: a shallow one at *r* ≈ 8 (inset, cyan line) and a more pronounced one at *r* ≈ 20.5.

By further increasing *T* (Figure 5, point *H* and further), the atomic ejection rate is not compensated by an equal rate of atom intake. The sphere collisional section becomes very small and is unable to sustain the two rates equality, making the liquid sphere disappear. The transition process ends with the box containing only a one-phase gas. The entire process is summarized in Table 5 and Figure 5, showing that initially, the transition occurs from solid to gas, and at the end, from liquid to gas, indicating that the system crosses a triple point line or region in the intermediate stages (Figure 5, narrow gray area).

**Table 5:** Summary of the Isobaric Transition Process and Equilibrium States

D30 nanoparticle, pressure *p* = 0.1					
temperature	*T* < 0.35	0.35 ≤ T < 0.5	0.55 ≲ T ≲ 0.6	0.6 ≤ T < 0.9	T ≥ 0.9
equilibrium	solid	solid–gas	solid–liquid–gas	liquid–gas	gas

Importantly, the solid-to-gas and the liquid-to-gas transitions occur at finite temperature intervals Δ*T*_SG_, Δ*T*_LG_ unlike in homogeneous systems, where transitions happen at constant temperature. Similar Δ*T* intervals have been observed during phase transitions of nanoparticles in previous computational studies. These include MD simulations of LJ systems,^62^ Au,^48,63^ Ag,^64^ Cu^65^ NPs, as well as QM/MM simulations of Ar.^66^ Experimental results for Ar,^67^ Na,^68,69^ and Ag^30,44^ also report phase transitions where physical observables do not exhibit sharp changes. Instead, these changes occur over a temperature range, referred to as the melting range or multistage transition.^70^ The broad peaks observed in calorimetry experiments for metals such as Pb,^71^ Al,^72^ and Sn^73^ can also be attributed to a Δ*T* interval. The width of these peaks reflects the range over which the phase transition occurs, and identifying a specific temperature within that range is a matter of convention. The association of the peak position of such physical observable (in most cases, the heat capacity *C*_p_(*T*)) with the transition temperature is a legacy of the standard thermodynamics model, which is strictly valid for homogeneous systems.

#### 2.2.2 Isobaric Transition in Nanoparticles of Different Size

After this analysis, we investigate smaller and larger NPs to highlight how the NP size affects the intensive variables profiles. NPs up to *d* = 90 diameter have been considered (see Table 3). In particular, by considering very large NPs, we will arrive at the important conclusion that the behavior of homogeneous systems *is not* obtained by indefinitely extending the size of FNH systems.

**Figure 8:**
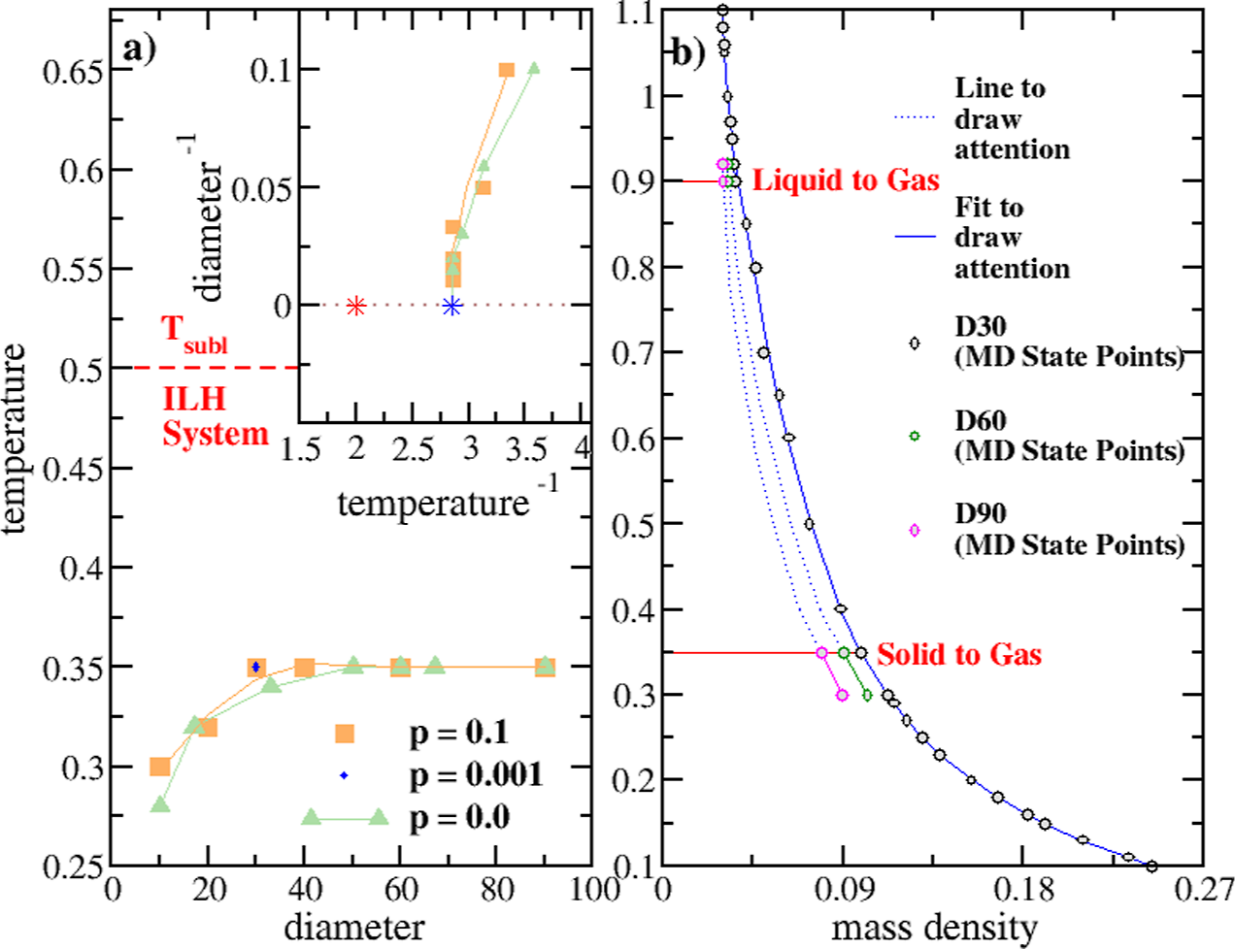
(a) Scaling of sublimation temperature. (b) Isobars for D30, D60, and D90 samples. Panel (a) has been adapted with permission from “Bose, S. Molecular Dynamics Modelling of Thermostatic Properties of Nanoparticles (2020)” (ref (52)). Copyright 2020 https://zenodo.org/records/3911784.

First, the size influences *T*_SG_, the temperature at which the first atom is ejected from the solid sphere, indicating the onset of sublimation (Figure 5). Figure 8a shows two sets of results, for *p* = 0.0 and *p* = 0.1. Except for small sphere diameters, the results for both pressures are similar, exhibiting an asymptotic behavior toward a limiting value of *T*_SG_ ≈ 0.35, which is reached for *d* ≈ 40. The inset of Figure 8a shows the same results but with *d*^–1^ plotted versus . This representation makes it clearer that the D60 and D90 spheres resemble very large FNH systems (*d* ≫ 1) and that *T*_SG_ ≈ 0.35 is the limiting value for large FNHs (blue star). Figure 8a also reports the sublimation temperature of the corresponding infinitely large homogeneous system (ILH), T_SG_^ILH^ = 0.5 (red dashed line, red star), as determined by van der Hoef at *p* < 10^–5^.^74^ Analogous simulations reported T_SG_^ILH^ = 0.5 for *p* = 0^75^ and T_SG_^ILH^ = 0.55 for *p* ≈ 6 × 10^–5^.^76^ The sublimation temperature difference between ILHs and FNHs, Δ*T* = T_SG_^ILH^ – *T*_SG_, demonstrates that the limiting homogeneous system value is not attained by the FNHs. Consequently, it is not possible to reconcile ST model results with the thermodynamics of real nonhomogeneous finite systems.

*T*_LG_ denotes instead the temperature at which the very last portion of the sphere, still in stable equilibrium with the gas, disappears. We could not determine its value with the same accuracy as *T*_SG_^11^. However, we expect the D30, D60, and D90 spheres to have a similar *T*_LG_ ≈ 0.9 (Figure 8b), as in the last stages of the transition, all three systems are liquid, with small droplets in equilibrium with their own gas phase. Additionally, the three systems are in close thermodynamic states, as shown by the isobars in Figure 8b and the similar *g*(*r*) plots (see Section S3 and Figure S3). Thus, for *d* ≥ 30, *T*_LG_ = 0.9 can be chosen as the size-independent temperature for the final transition.

This analysis leads to several conclusions for FNHs at moderate pressure: (i) the initial solid-to-gas transition temperature *T*_SG_ reaches a constant, limiting value of 0.35 for diameters *d* ≳ 40, differing from the one of homogeneous systems; (ii) the final liquid-to-gas transition temperature *T*_LG_ has small variations with the diameter; (iii) between *T*_SG_ and *T*_LG_, and due to the moderate pressure, all systems cross a triple point line or region; (iv) in FNHs, transitions happen during *finite* temperature intervals Δ*T*_SG_ and Δ*T*_LG_, encompassing a total interval Δ*T*_tr_ = *T*_LG_ – *T*_SG_ (see Figure 5); (v) as a consequence, FNH phase diagrams are characterized by phase equilibrium *regions*, rather than lines as in homogeneous systems. Finally, (vi) the FNH equilibrium between two phases in contact is determined by spatially dependent intensive variables in the interfacial region and not by bulk extensive variables that can only characterize on average the entire system. These considerations lead us to conclude that, very generally, the limiting behavior of FNH systems when indefinitely extending their size *will not* approach that of the corresponding homogeneous systems.^12^

Our analysis in terms of profiles serves to further interpret experimental data about argon,^67^ gold,^39,40,69,77−80^ silver,^41,44^ copper,^81^ tin,^82^ and sodium^69,83^ nanoparticles, for which theoretical approaches^80,84,85^ and simulations^79,86−90^ have been developed and performed. Several reviews on these systems^70,84,85,91^ indicate that while the models describing phase transitions (each based on specific approximations) are generally valid, none fully capture all experimental findings.

## 3 Conclusions

In this work, we addressed the thermodynamic stability mechanisms of finite nonhomogeneous systems (FNHs) using classical molecular dynamics (MD) simulations, choosing Lennard-Jones spherical nanoparticles as prototypical systems. Analyzing the MD trajectories, we constructed radial profiles made of intensive variables which, due to nonhomogeneity, turned out to be spatially dependent. These profiles included, most notably, an average potential energy (PE) *e*_pot,k_, an instantaneous maximum of the atomic kinetic energy (KE) max(*e*_kin*,*k_), and a force profile (*F*_k_). We could explain stability and equilibrium by comparing the PE and KE profiles and analyzing the structures of the force profile arising from the sphere’s nonhomogeneity. As a function of temperature, each profile highlights different aspects of the stability; e.g., the potential energy shows where the atoms move, the vibrational and/or diffusional character of their motion in the nanoparticle inner core/interface, and the system volatility. We find that the stability and equilibrium criteria in FNHs are not governed by extensive variables characterizing the bulk region but by spatially dependent intensive variables at the interface, in contrast to the standard thermodynamics (ST) model, which assumes homogeneous systems.

Next, we investigated two-phase equilibria and phase diagrams during isobaric phase transitions (PTs), considering nanoparticles with varying diameters as a function of temperature. By combining the insights provided by the radial profiles with the analysis of atomic trajectories, we observed how, during the PTs, atoms ejected from/returning to the sphere establish a dynamic equilibrium with the solid or liquid interface of the sphere. As the temperature increases, nanoparticles undergo a solid-to-gas sublimation, pass through a three-phase region, and transition to a liquid–gas phase before completely vaporizing. These transitions were computed for increasing diameters *d*, allowing the following conclusions: (i) for each *d*, the transitions occur within finite intervals Δ*T*_SG_ and Δ*T*_LG_, in contrast with the ST model, for which homogeneous system transitions occur for Δ*T* = 0; (ii) beyond a certain diameter, *T*_SG_ and *T*_LG_ asymptotically approach limiting values, which do not converge to homogeneous system values T_SG_^ILH^ and T_LG_^ILH^. Hence, as this happens even for very large systems (for *N* → ∞), homogeneous systems are not the correct limit of FNHs, showing that finite-size effects and the presence of boundaries cannot be neglected when dealing with real, finite systems.

## 4 Methods

### 4.1 Software

MD simulations were performed using the LAMMPS software.^23^ Visualizations of the atomic snapshots were created using OVITO.^92^

### 4.2 Sample Preparation and Stability Check

LJ spheres with different diameters were obtained as follows: two preliminary LJ finite crystals were constructed, each with the same number of atoms. The first was in its global minimum configuration, as determined by density functional theory,^93^ and showed a faceted solid structure. The second was spherical and obtained by cutting a bulk system, followed by a structural minimization using the conjugate gradient method implemented in LAMMPS,^23^ and subjected to an annealing cycle with heating and cooling up to *T* = 0.3 and *p* = 0. Each system was placed in a large simulation box, and *NVT* simulations were conducted by gradually increasing the temperature in increments of Δ*T* = 0.02. Production runs began only after confirming the invariance of all profiles discussed in this work. The invariance confirmed that the simulation time exceeded all relevant relaxation times, specifically: τ_mech_ ≪ τ_therm_ ≪ τ_chem_, corresponding to momentum, energy, and mass transfer, respectively, indicating equilibrium^35^ of intensive variables (discussed further in the next subsection). At very low *T*, the total energy and spatial profiles of the two systems were significantly different. However, at T* ≈ 0.1, both quantities converged and became statistically equivalent. For *T* > 0.1, system snapshots confirmed the formation of small facets in both configurations. This methodology ensures that, for *T* beyond a specific T*, the system derived from the spherical cut evolves comparably to the one initially at the global minimum. Thus, all configurations generated by our MD simulations correspond to the evolution of a system initially at its global minimum.

### 4.3 MD Setups and Parameters

All setups and parameters used are summarized in Tables 1, 2, and 3. Two MD setups have been established: one to investigate the one-phase stable systems and the other to study the equilibrium between two phases in contact with each other (see Table 1). Unless specified otherwise, heating runs consisted in 2 × 10^6^ steps, equilibration runs in 4 × 10^6^ steps, and production runs in 2 × 10^6^ steps. For two-phase thermodynamic states, the equilibration runs, on average, extended for 1 – 2 × 10^7^ steps (with longer duration for larger spheres, i.e., D60 and D90). A larger time step was used for two-phase states, after initial 5 × 10^6^ equilibration steps, to expedite the atom ejection process and attain well-equilibrated states characterized by a zero slope in *N*_i_ (where i is the phase index).

### 4.4 Equilibration Criteria

Two phases of a system are in equilibrium when the chemical potential μ is identical in both phases or, equivalently, when the molar (or per particle) free energy is the same for each phase. This implies that moving an atom from one phase to the other does not change the overall total free energy of the system. At equilibrium, for every atom that makes a transition from one phase to the other, a reverse transition occurs, and the net flux of atoms is zero. To verify that μ is equal, standard thermodynamics (ST) requires the evaluation of the free energy/chemical potential of the two separate phases. Only in this way, the ST approach identifies the thermodynamic conditions under which the phases are in equilibrium. Several methods, such as umbrella sampling and thermodynamic integration,^94^ are available for evaluating free energies. However, these methods cannot directly determine the net flux of atoms. On the other hand, a thermodynamic approach to nonhomogeneous systems, based on MD, makes it possible to study two phases in direct contact. In this approach, the equilibrium is determined by directly evaluating the net flux of atoms between the phases. When this flux is zero, the phases are in equilibrium, and their chemical potentials and (molar) free energies must be equal, even if their explicit values are not calculated. This is the approach adopted in this work, which focuses on finite nonhomogeneous systems (FNHs). We use MD to determine equilibrium by identifying when the net flux of atoms is zero, without explicitly evaluating chemical potentials and free energies. In FNHs, verifying the state of equilibration requires extra care as compared to homogeneous systems because the three thermodynamic equilibrium conditions (mechanical, thermal, and chemical) have their own relaxation times τ_mech_, τ_therm_, and τ_chem_. As for dense phases, it is known that τ_mech_ ≪ τ_therm_ ≪ τ_chem_,^35^ it follows that in FNHs, attaining equilibrium must be verified by ensuring that the mass flux (having the longest τ = τ_chem_) reaches a zero value. This applies both to one-phase (flux inside the nanoparticle) and to two-phase states (flux throughout the entire system).

Based on these criteria, equilibrium in one-phase states is indicated not only by the convergence of the system’s total energy (or the minimization of other thermodynamic potentials) but also by the absence of atomic motion between inner and surface/interface regions. In our simulations, we confirm mechanical equilibrium as the radial momentum flux (and the slope of kinetic energy) is essentially zero, as shown in Figure 1b. Thermal equilibrium is established when the slope of the total energy is ≤10^–10^. To verify chemical equilibrium, we monitor the invariance of the density profile after the system has reached a well-equilibrated state. Despite the distribution of potential energy within the system (see Figure 1a), we find that the density profile remains stable within the simulation time with no net mass flux (see Figure 3b).

For two-phase states, in addition to the above conditions (which are met), it is crucial to monitor the number of atoms in each phase, which should remain constant at equilibrium. In our simulations, upon reaching equilibrium, the number of atoms in each phase fluctuates around a constant mean value (see Figure 4a). These fluctuations are characteristic of a dynamic equilibrium, and our two-phase state remains stable within the simulation time.

To illustrate how the systems approach equilibrium with time, for the D30 two-phase state, the slope values of the equilibration metrics were computed over distinct time intervals (see Table 6 for quantification). The kinetic energy converges almost instantaneously, whereas the total energy (sum of kinetic and potential contributions) requires a time interval nearly 2 orders of magnitude longer to achieve an effectively zero slope. The total energy converges more slowly than the kinetic energy since the potential energy reflects the slower structural minimization process. The slope corresponding to the number of atoms in each phase (*N*_sph_ or *N*_gas_) converges even more slowly, taking the longest time (for this state point ≈14 M time steps) since it reflects much slower structural reorganization processes such as diffusion and ejection. These observations clearly demonstrate that the expected hierarchy of relaxation times τ_mech_ ≪ τ_therm_ ≪ τ_chem_ is maintained.

**Table 6:** Time Evolution of the Slope Values for the Equilibration Metrics for the D30 Two-Phase System at *T* = 0.5 and *p* = 0.1 (Point *D* in Figure 5)^a^

	time steps			
	0 – 5 × 10^4^	5 × 10^4^ – 2.5 × 10^5^	2 – 3 × 10^6^	1.4–1.8 × 10^7^
kinetic energy	1.65 × 10^–7^	1.24 × 10^–10^		
total energy	7.04 × 10^–7^	2.15 × 10^–8^	8.87 × 10^–10^	
*N*_sph_ or *N*_gas_	1.98 × 10^–1^	9.97 × 10^–2^	2.71 × 10^–3^	5.48 × 10^–10^

a Equilibrium is considered achieved when the slope reaches ≤10^–10^, i.e., effectively statistically zero.

### 4.5 Distinguishing Atoms in Different Phases

The number of atoms in the two phases, *N*_i_ (i being the phase index), is shown in Figure 4a. To distinguish N_sph_ from *N*_gas_, atoms are classified as belonging to the gas phase if the distance to their nearest neighbor is *d*_neigh_ ≥ 1.7 and to the solid/liquid phase if *d*_neigh_ < 1.7. The choice of the 1.7 threshold is a balanced measure to avoid miscounting gas-phase dimers or trimers as a part of the sphere. Essentially, it is smaller than the typical distances within gas-phase dimers or trimers. However, this threshold could be set higher if not for the presence of these dimers/trimers as the distance between the monomers in the gas phase is significantly larger than 1.7.

### 4.6 Inert Gas

For two-phase thermodynamic states at *p* > 0, introducing inert gas atoms is necessary to maintain the chosen pressure. The number of inert gas atoms incorporated into the D30, D60, and D90 systems was selected to maintain a comparable total mass density in the simulation box (comprising both FNH sphere atoms and inert gas atoms) across the studied systems, at *T* = 0.3 and *p* = 0.1. Determining the number of inert gas atoms serves two purposes: (i) to ensure the simulation box is sufficiently large, ideally with a side length at least twice the sphere diameter, to prevent interactions between the sphere’s image replicas and (ii) to maintain the chosen pressure without the simulation box shrinking in a manner that would violate purpose (i). Given the inclusion of the inert gas, the mass density reported in Section 2.2 always reflects the total mass density of the simulation box. Consequently, this density cannot be directly compared with the densities used to illustrate the isobars of the corresponding homogeneous systems in the literature.^95−97^

### 4.7 Thermostat and Barostat

The damping coefficients for the Nosé-Hoover thermostat and barostat were set at 100 and 500 × time step size, respectively.

### 4.8 Ensuring Zero Momentum of the Whole Sphere

For simulating one-phase thermodynamic states, the use of fixed boundary conditions requires net zero linear momentum of the whole sphere, to prevent the system from drifting toward the simulation box boundaries.

### 4.9 Units

All quantities are expressed in reduced Lennard-Jones units, and in the text/figures, they appear without units.

## Supporting Information Available

Expressions for radial profiles, such as the number density profile, the diffusing atom fraction, the potential, kinetic energy, maximum kinetic energy, force profiles, and the pair distance distribution; details on the quasi-ideal nature of the inert gas included in the calculations; demonstration showing that D30, D60, and D90 nanoparticles at *T* = 0.9 are in similar thermodynamic states; and convergence of various profiles with respect to the LJ interaction cutoff (PDF)

## Footnotes

1 This stems from pair distribution functions^2^ analyses, showing that, at high temperature, the gas–gas distribution function and the inert container wall–gas distribution become indistinguishable.

2 Systems are assumed uncharged, chemically inert, without long-range interactions and not acted on by electric, magnetic, or gravitational fields.^3^

3 *S* is an extensive function of the extensive variables *E* and ***X*** when *S*(λE, λ***X***) = λS(*E*, ***X***).

4 In standard thermodynamics, the spatial dependence of “intensive” and “macroscopic” variables means they are not strictly intensive or extensive. However, for simplicity, we will continue to use these terms.

5 Our systems are considered stable within the duration of our MD simulations. For each system, the simulation time is chosen to significantly exceed *all* relevant systems’ relaxation times, in analogy with what happens during experimental observations (see Section 4.4 for further discussion).

6 All data/results are expressed in reduced LJ units;^21−23^ in the following, they will appear as pure numbers.

7 The KE distribution broadening is given by |*max*(*e*_kin,k_) – ⟨*e*_kin,k_⟩|. The slope is due to a rise in the particle count within shell k as a function of *r*, resulting (on average) in a higher KE maximum derived from a larger statistical sample.

8 This value has been adopted since surface atoms can exhibit msd values orders of magnitude larger than those of bulk atoms.^49−52^ In our simulation, as shown in Figure 3a, msd_bulk_ ≈ 0.01 (blue line), while msd_surface_ ≥ 1.0 (red line).

9 Note that, from our definitions, . Moreover, *F*_k_ is not even the usual gradient of the potential energy. *F*_k_ = −d*e*_pot,k_/d*r*_k_ stems from the difference in the PE energy minima between contiguous shells k and k + 1. In the finite chain example, it accounts from the different minima of the parabolas close to the chain edge.

10 Only recently, efforts have been made to identify PDD universal features that distinguish the phases of NPs.^60^ In the solid phase, the PDD exhibits strong oscillations, with at least the first peak going to zero. In the liquid phase, PDD oscillations do not go to zero between peaks, and they decay exponentially.

11 This is due to a very heavy computational cost, as, e.g., observing the complete evaporation of the D90 liquid droplet at *T* = 0.9 requires >40 M time steps (the D90 sample has 1.85 M atoms).

12 In contrast, the thermodynamics of small systems developed by T.L. Hill^12^ suggests that the above-mentioned temperature intervals should approach zero in the limit of large systems.
