# Supplementary material for: Thermodynamic States in Nonhomogeneous Systems: From Nanoscale to Macroscale
Source: ACS Omega. 2025 Apr 9;10(15):15321–33. doi: 10.1021/acsomega.4c11379 (PMC12019454; doi:10.1021/acsomega.4c11379)
Supplement: Supplementary file 1 — ao4c11379_si_001.pdf [file ao4c11379_si_001.pdf]

# Supporting Information

# Thermodynamic states in non-homogeneous systems: from nanoscale to macroscale

**Sankhadeep Bose[†], Andrea Floris[*,‡], Mangaiyarkarasi Rajendiran[¶], and Bruno D'Aguanno[‡]**

[†]*School of Mechanical Engineering, Vellore Institute of Technology, Vellore, 632014, Tamil Nadu, India*

[‡]*Department of Chemistry, School of Natural Sciences, University of Lincoln, Brayford Pool, LN6 7TS, Lincoln, United Kingdom*

[¶]*Centre for Nanotechnology Research, Vellore Institute of Technology, Vellore, 632014, Tamil Nadu, India*

**E-mail:** afloris@lincoln.ac.uk

# 1 Expressions for radial profiles

## 1.1 Number density profile

The number density profile $\rho_k$ serves as a descriptor for the spatial distribution of atoms within a system. It quantifies the average number of atoms within spherical shells of a given thickness around a reference point (center of mass) in the system. The profile is derived from the atomic positions, considering volumes defined by spherical shells extending from radius $r_k$ to $r_k + \delta r$. Mathematically, $\rho_k$ is expressed as:

$$\rho_k = \frac{\langle N_k \rangle}{\Delta V_k}. \tag{1}$$

Here, $\langle N_k \rangle$ represents the MD time-averaged count of atoms located within the spherical shell volume $\Delta V_k = \frac{4}{3}\pi \left[ (r_k + \delta r)^3 - r_k^3 \right]$ at a radial distance $r_k$ from the reference point (center of mass).

## 1.2 Diffusing atom fraction

The diffusing atom fraction $x_D$ is a parameter for assessing the mobility of atoms within a system. It measures the fraction of atoms that exhibit diffusive behavior beyond a defined threshold within a specified region or shell of the simulation domain. The diffusing atom fraction is calculated as:

$$x_D = \frac{\left\langle N_k^D \right\rangle}{N_k} \text{ such that } \left\langle N_k^D \right\rangle : \sqrt{(msd)_{i \in k}} \geq 1.0\sigma \tag{2}$$

where $\left\langle N_k^D \right\rangle$ the MD time-averaged number of atoms classified as 'diffusing' within the shell $k$. An atom is considered to be diffusing if its root mean squared displacement satisfies the condition $\sqrt{(msd)_{i \in k}} \geq 1.0\sigma$. The selection of $1.0\sigma$ as the threshold for distinguish-

ing diffusive from vibrational atoms is influenced by the Lindemann-like criterion proposed elsewhere,[52] when their displacement exceeds their equilibrium lattice constant ($\approx 1.0\sigma$).

## 1.3 Potential energy profile

The potential energy profile $e_{pot,k}$ quantifies the average potential energy (per atom) experienced by atoms within a shell $k$. The calculation of $e_{pot,k}$ incorporates not only the interactions among atoms within the same shell but also their interactions with all atoms in the system that fall within a predefined interaction cutoff distance. The equation for the potential energy profile is given by:

$$e_{pot,k} = \left\langle \frac{1}{N_k} \sum_{i \in k} \sum_{\substack{j=1 \\ j \neq i}}^{N} e_{LJ}(r_{ij}) \right\rangle \tag{3}$$

where $e_{LJ}(r_{ij})$ denotes the potential energy between a pair of atoms $i$ and $j$, calculated using the Lennard-Jones interaction based on the distance between the atoms. The double summation iterates over all atom pairs where $i$ is an atom within shell $k$, and $j$ represents all atoms in the system (within the cutoff distance), excluding self-interactions. $\langle \cdots \rangle$ denote the time average over the duration of the MD simulation.

## 1.4 Kinetic energy profile

The kinetic energy profile $e_{kin,k}$ describes the average kinetic energy per atoms within the shell $k$. The profile is computed as follows:

$$e_{kin,k} = \left\langle \frac{1}{N_k} \sum_{i \in k} \frac{m v_i^2}{2} \right\rangle \tag{4}$$

where, $m$ is the atomic mass and $v_i$ is the velocity of the $i$-th atom within the shell $k$. $N_k$ is the total number of atoms in the shell and $\langle \cdots \rangle$ indicates time averaging over the MD simulation.

## 1.5 Maximum kinetic energy profile

The maximum kinetic energy profile $\max(e_{kin,k})$ characterizes the peak kinetic energy attained by atoms within a shell $k$, throughout the entire MD simulation time. It is expressed as:

$$\max(e_{kin,k}) = \max_{i \in k} \left( \frac{m v_i^2}{2} \right) \text{over all time steps} \tag{5}$$

The operation $max_{i \in k}$ computes the value of the maximum kinetic energy achieved among all the atoms in shell $k$ at a single time step, followed by an outer maximization over the full simulation duration, to capture the absolute maximum kinetic energy recorded throughout the simulation time.

## 1.6 Force profile

The force profile $F_k$ is derived from the spatial gradient of the potential energy profile (see Eq. 3), measuring the force acting on atoms within a shell $k$. Its expression reads:

$$F_k = -\frac{de_{pot,k}}{dr_k}. \tag{6}$$

## 1.7 Pair distance distribution

The pair distance distribution $PDD$ is a measure of the distribution of distances between pairs of atoms in a system, without incorporating normalization by the ideal gas distribution.

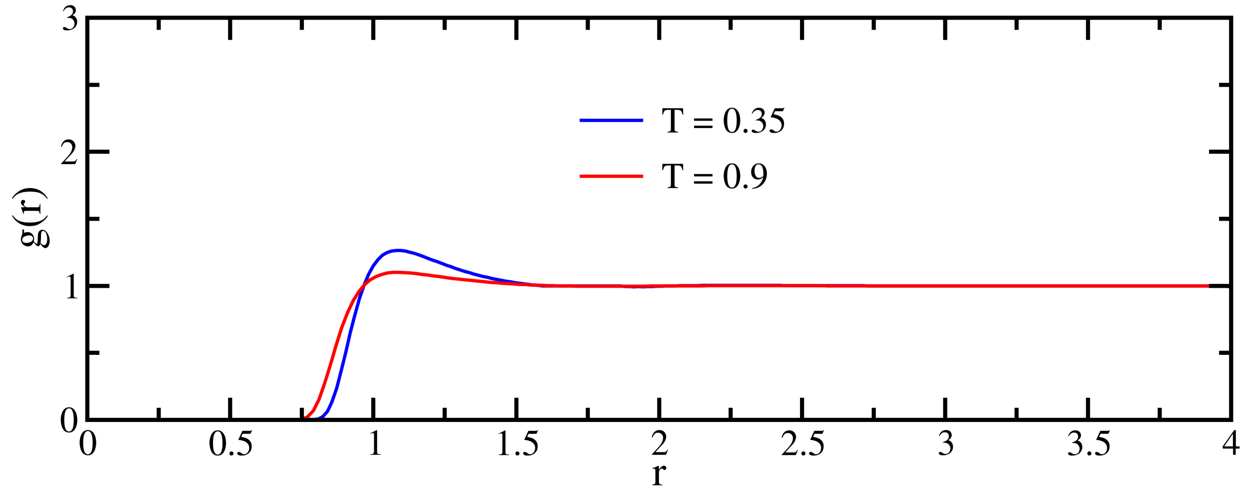


Figure S1: Pair distribution function $g(r)$ of the inert gas at $T = 0.35$ (blue) and $T = 0.9$ (red).

It is expressed as:

$$PDD = \left\langle \frac{1}{N} \sum_{\substack{i=1 \\ i \neq j}}^{N} \delta\left(r - |\vec{r_i} - \vec{r_j}|\right) \right\rangle, \tag{7}$$

where $r$ is the distance between the two atoms, $\vec{r_i}$ and $\vec{r_j}$ are the position vectors of the $i$th and $j$th atoms, and $\langle \cdots \rangle$ denote the time average over the MD simulation.

## 2 Quasi-ideal nature of the inert gas

In this section, we demonstrate the (quasi-)ideal nature of the inert gas used in our study to achieve two-phase equilibrium. We also show that the inclusion of this inert gas only slightly modifies the quantitative results of the radial profiles for intensive variables, while the qualitative characteristics of these profiles were entirely preserved.

Fig. S1 shows the pair distribution functions, $g_{inert} - g_{inert}$, evaluated at $T = 0.35$ and $T = 0.9$, spanning the entire temperature range of interest for our simulations of two-phase equilibrium systems. The $g(r)$, irrespective of temperature, exhibits only a very minor peak, followed by a complete disappearance of correlation for larger values of $r$ i.e. $g(r) - 1 \rightarrow 0$.

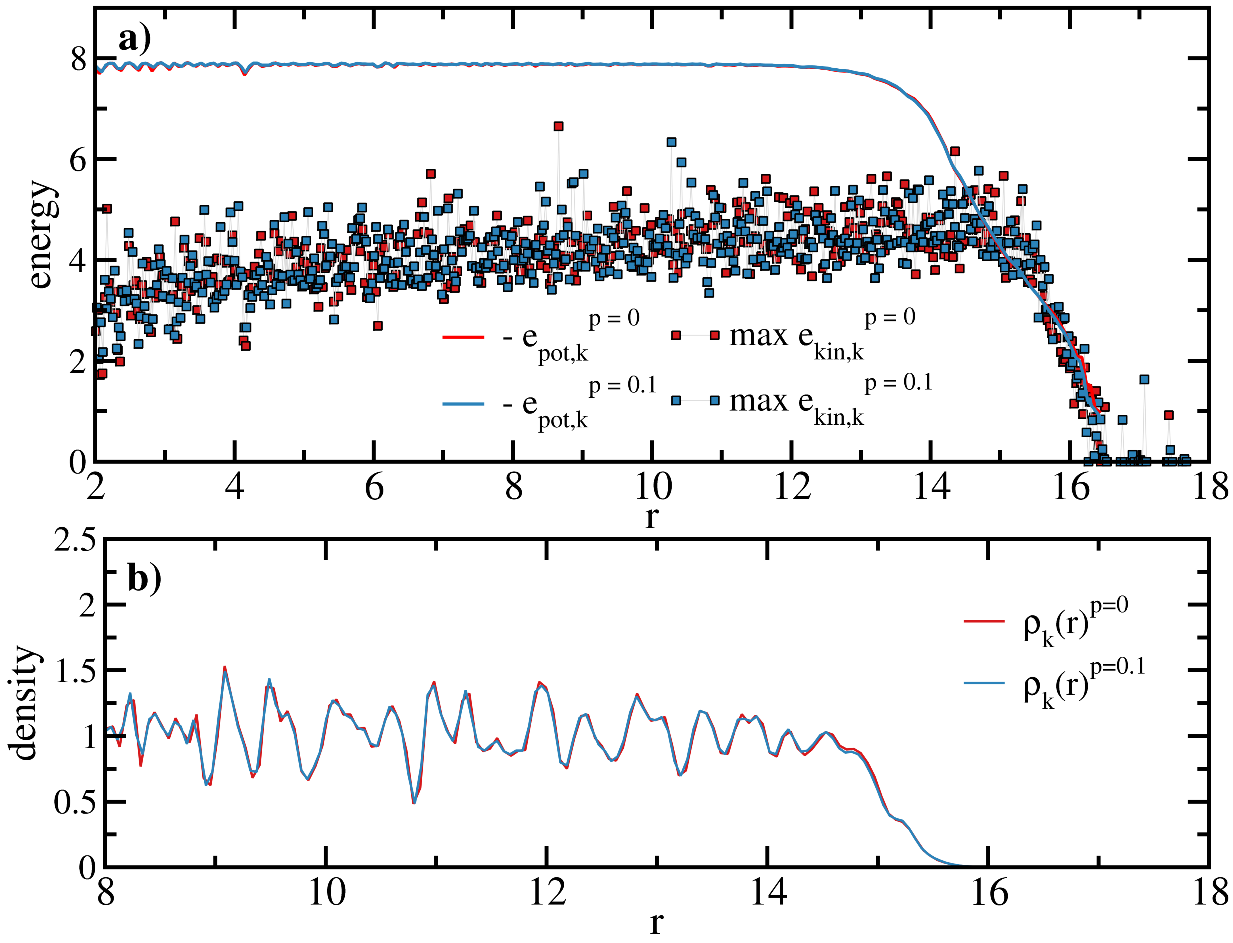


Figure S2: Effect of inert gas on the radial profiles of a D30 nanoparticle. Panel (a): potential energy $-e_{pot,k}$ (solid lines) and maximum kinetic energy profiles $\max(e_{kin,k})$ (squared points) in vacuum ($p = 0$, red plots) and in the presence of an inert gas ($p = 0.1$, blue plots). Panel (b): density profiles $\rho_k$ under the same conditions.

This behavior is what is expected in the ideal gas limit (for example, see Fig. 4.8 in[2]).

Fig. S2 further explores the effect of the inert gas on the radial profiles. It is evident that the inert gas does not alter the features in any significant manner. For both the potential energy $e_{pot,k}$ and the maximum kinetic energy $\max(e_{kin,k})$ profiles, the qualitative characteristics remain similar. This also includes conditions required for the atoms to leave the surface. Similarly, the density profiles $\rho_k$ remain unaffected in both the homogeneous internal region and the non-homogeneous surface regions, with the spatial distribution remaining fundamentally unchanged.

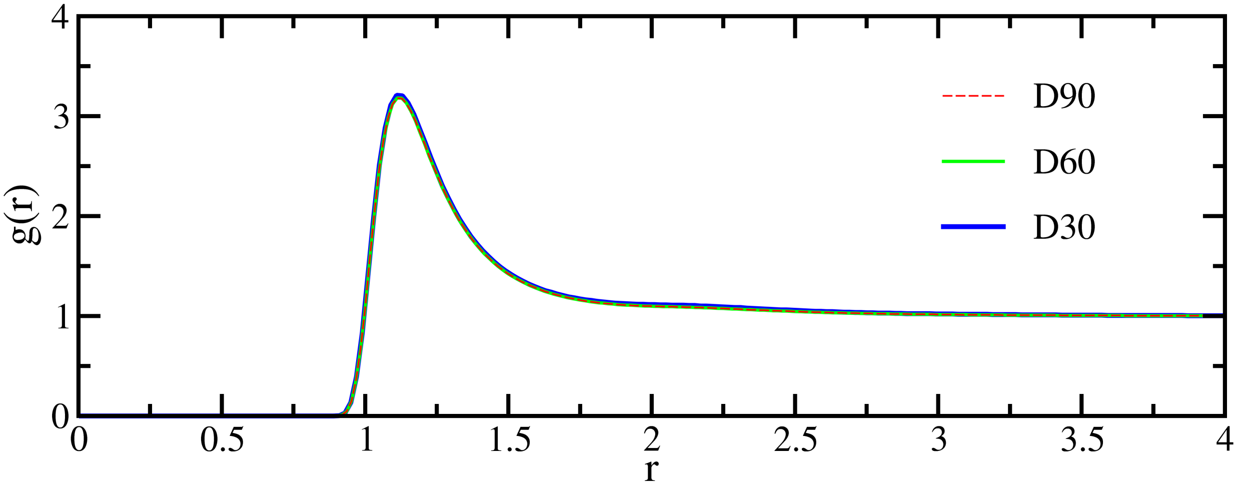


Figure S3: Pair distribution function $g(r)$ of D30 (blue solid line), D60 (green solid line) and D90 (red dashed line) nanoparticles at $T = 0.9$.

## 3 $g(r)$ of D30, D60 and D90 nanoparticles at $T = 0.9$

In this section, we establish that at $T = 0.9$, the systems - D30, D60, and D90 are in similar thermodynamic states. This conclusion is supported by the analysis of the pair distribution functions ($g_{NP} - g_{NP}$) for D30, D60, and D90 at $T = 0.9$ ($p = 0.1$), as shown in Fig. S3. The pair distribution functions, $g(r)$, exhibit inherently identical characteristics across the systems, consistent with those observed in a low-density limit gas.[2] This includes the presence of only a single coordination sphere (with identical area under the peak) that rapidly decays, leading to a complete disappearance of correlation so that $g(r) - 1 \to 0$. Such observations indicate that the systems have microscopically identical structures and, consequently, occupy the same thermodynamic state.

## 4 LJ interaction cutoff convergence

In this section, we examine the convergence of the LJ interaction $r_{cut}$ by comparing the radial profiles of the one-phase D30 nanoparticle at $T = 0.35$ in vacuum ($p = 0$). Fig. S4

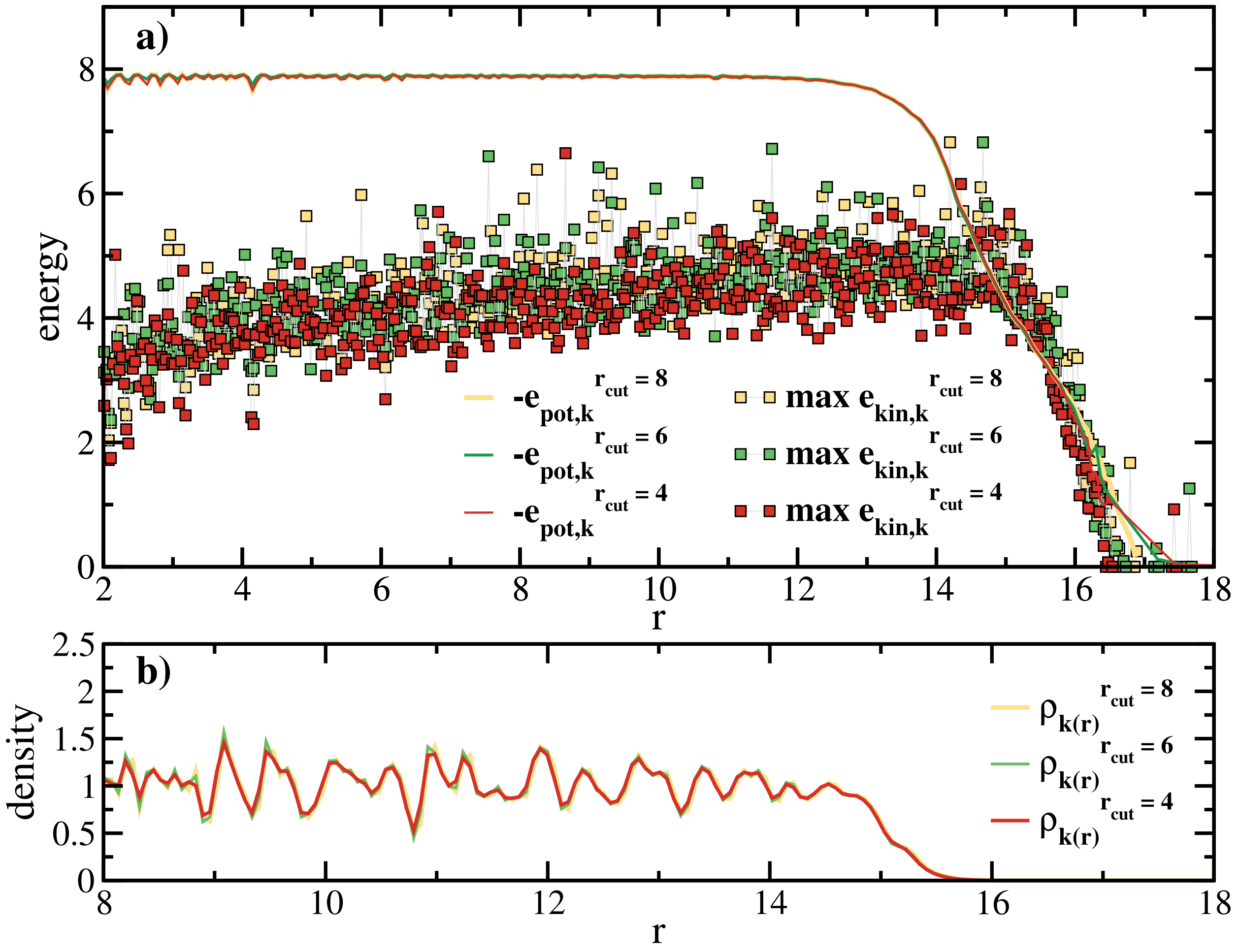


Figure S4: Radial profiles for a one-phase D30 nanoparticle at $T = 0.35$ using three LJ interaction $r_{cut}$: 4.0 (red), 6.0 (green), and 8.0 (yellow). Panel (a): Potential energy $-e_{pot,k}$ (solid lines); maximum kinetic energy $\max(e_{kin,k})$ (squared points). Panel (b): Density $\rho_k$ profiles.

shows the potential energy $e_{pot,k}$, the maximum kinetic energy $\max(e_{kin,k})$ and the density $\rho_k$ profiles for three chosen $r_{cut}$: 4.0, 6.0, and 8.0. All profiles overlap and are statistically indistinguishable. Consequently the three criteria (see subsection 2.1) that an atom must meet to leave the nanoparticle remain the same for all these $r_{cut}$ values. This behavior can be attributed to the rapid $r^{-6}$ decay of the LJ potential, which makes force contributions beyond a certain distance negligible. Indeed, even at a $r_{cut} = 2.5$ "the potential is merely 1.6% of its value at the minimum".[26] We therefore conclude that $r_{cut} = 4.0$ is sufficiently large to achieve convergence of the radial profiles while leaving the phase diagram unaffected.
